# Finding the balance between person-centred and treatment-centred discussions in advance care planning—a qualitative analysis of conversations within the MUTUAL (Multidisciplinary Timely Undertaken Advance Care Planning conversations) intervention using a narrative analysis

**DOI:** 10.1093/ageing/afae020

**Published:** 2024-03-06

**Authors:** Eline V T J van Lummel, Larissa Ietswaard, Marloes Rigter, Dave H T Tjan, Johannes J M van Delden, Megan Milota

**Affiliations:** Department of Intensive Care, Gelderse Vallei Hospital, Ede, Netherlands; Department of Bioethics and Health Humanities, Julius Center for Health Sciences and Primary Care, University Medical Center Utrecht, Utrecht, Netherlands; Department of Bioethics and Health Humanities, Julius Center for Health Sciences and Primary Care, University Medical Center Utrecht, Utrecht, Netherlands; Department of Bioethics and Health Humanities, Julius Center for Health Sciences and Primary Care, University Medical Center Utrecht, Utrecht, Netherlands; Department of Intensive Care, Gelderse Vallei Hospital, Ede, Netherlands; Department of Bioethics and Health Humanities, Julius Center for Health Sciences and Primary Care, University Medical Center Utrecht, Utrecht, Netherlands; Department of Bioethics and Health Humanities, Julius Center for Health Sciences and Primary Care, University Medical Center Utrecht, Utrecht, Netherlands

**Keywords:** advance care planning, person-centred care discussions, treatment-centred care discussions, narrative analysis, qualitative research, older people

## Abstract

**Introduction:**

Advance care planning (ACP) aims to create conditions for more person-centred care. We aimed to explore variations in person-centred care discussions and treatment-centred care discussions within ACP conversations in the Multidisciplinary Timely Undertaken Advance Care Planning (MUTUAL) intervention and how person-centred care discussions could be encouraged. The MUTUAL intervention consists of the following: (i) timely patient selection, (ii) the patient and healthcare professionals preparing for the conversation, (iii) a scripted ACP conversation in a multidisciplinary setting and (iv) documentation.

**Methods:**

We conducted a narrative analysis of ACP conversations. A narrative summary template was created and used to analyse 18 audio-recordings.

**Results:**

We noticed variations in person-centred and treatment-centred focus within the ACP conversations. We identified three important strategies that facilitated person-centred care discussions within ACP conversations. First, healthcare professionals’ acceptance that ACP is an individual process. We believe it is important that healthcare professionals recognise and accept where the patient is in his or her individual ACP process; not making decisions right away can also be part of a decisional process. Secondly, exploring the underlying motivation for treatment wishes can give insights into patient’s wishes, values and needs. Lastly, healthcare professionals who demonstrated an adaptive, curious and engaged attitude throughout the ACP process achieved more person-centred ACP conversations. This coincided with elaborating on the patient’s emotions, fears and worries.

**Conclusion:**

Person-centred and treatment-centred focus varied within the ACP conversations in the MUTUAL intervention. Certain strategies by healthcare professionals facilitated a more person-centred focus.

## Key Points

- It is important that healthcare professionals recognise and accept where the patient is in his or her individual advance care planning process
- Not making decisions right away can also be part of a decisional process
- Exploring the underlying motivation for patient treatment wishes can give insights into patient’s wishes, values and needs
- Addressing treatment preferences can serve as an avenue to elaborate on patient values and goals of care
- Demonstrating an adaptive, curious and engaged attitude can help achieve more person-centred advance care planning conversations

## Introduction

Advance care planning (ACP) is defined as ‘*enabling individuals to define goals and preferences for future medical treatment and care, to discuss these goals and preferences with family and healthcare providers and to record and review these preferences if appropriate’* [1]. According to Sudore *et al*. [2] ACP should prepare patients and proxies to participate with healthcare professionals in the decision-making process instead of focusing on advance treatment decisions. Therefore, the process of ACP can also be understood as a method to create conditions for person-centred care. The Institute of Medicine defined patient-centred care in 2001 as ‘*providing care that is respectful of and responsive to individual patient preferences, needs and values, and ensuring that patient values guide all clinical decision*’ [3].

The Multidisciplinary Timely Undertaken Advance Care Planning (MUTUAL) intervention was developed in 2018 [4]. An important element of the MUTUAL intervention is that a trained nurse starts the conversation, and is joined by the treating physician in the second part of the conversation. Earlier ACP interventions where only the facilitator was involved showed no effect on the quality of life [5]. Hence, we asked the treating physician to join the ACP conversation to support patients in the formulation and documentation of their treatment preferences. A physician joining the conversation might concretize patient preferences, bridging the gap between the patient’s thoughts and medical decision-making. At the same time, this approach might lead to a conversation primarily about medical treatments (hereafter referred to as ‘treatment-centred discussions’) as this tends to be physicians’ focus.

For example, in a survey among paediatricians by Fahner *et al*. [6], 60% of the paediatricians stated that ACP has to result in the documentation of a code status. Although their study showed that a broad range of ACP topics was discussed, the conversations seemed to concentrate on medical issues, potentially hampering an open exploration focused on person-centred care [6]. This is in line with Bernacki *et al*. [7] who discourage focusing on medical procedures in their narrative review analysing best practices for end-of-life communication.

Our multidisciplinary approach in the MUTUAL intervention might conflict with this advice. Hence, the question arises whether this multidisciplinary setting results in less attention for person-centred care discussions. Our first aim was to explore variations in person-centred care discussions and treatment-centred care discussions within the ACP conversations in the MUTUAL intervention.

Literature on how to encourage conversations about person-centred care within ACP is limited. Hence, our second aim was to determine how person-centred care discussions within ACP conversations could be encouraged.

## Methods

We conducted a qualitative analysis of ACP conversations that were part of a feasibility study of the MUTUAL intervention at Gelderse Vallei Hospital, a 300-bed, non-academic hospital in the Netherlands. The Consolidated Criteria for Reporting Qualitative Research (COREQ) were used to optimise reporting [8].

### Description of the MUTUAL intervention

The MUTUAL ACP intervention consists of four steps: timely patient selection, the patient and healthcare professionals preparing for the conversation, a scripted ACP conversation in a multidisciplinary setting (a trained nurse starting the conversation and the treating physician joining the conversation) and documentation of the conversation. An elaborate description of the development and feasibility study of the MUTUAL intervention can be found elsewhere [4]. A questionnaire was developed for patients to complete prior to the conversation. Patients were encouraged to complete this preparatory questionnaire and discuss the questionnaire with their proxies. A conversation manual was developed for the intervention and contains the following seven steps: (i) introduction of the topic; (ii) quality of life; (iii) goals of care; (iv) scenarios; (v) patient representative; (vi) conversation summary and conclusion and (vii) documentation. The facilitating nurse is allowed to deviate from the proposed topics and suggested structure. For example, the facilitating nurse can ask what the patient’s daily life looks like (referring to a question from the preparatory questionnaire) and where the patient places him/herself on the lifeline (a symbolic line with the start of the line representing birth and the end of the line representing death). Subsequently, the patient’s future goals and preferences, fears and worries and current experience of health can be discussed. This is often followed by discussing potential medical scenarios and discussing who should be the patient’s representative in case the patient is not able to express his/her wishes. In the second part of the conversation the physician joins the conversation, and the nurse facilitator gives a summary of the preceding conversation. Patients are encouraged to comment on or add information to the summary given by the nurse. In some cases, the nurse practices the summary with the patient before the physician enters. When joining the conversation, the physician is instructed to first listen to the summary and answer any questions. Subsequently the physician can ask clarifying questions in order to understand what is important to the patient. The physician is also instructed to further explore what potential treatment decisions can be made and documented.

### Participants and data collection

The study population consisted of patients attending the geriatric or pulmonology outpatient clinic of the Gelderse Vallei Hospital between March 2018 and April 2018. All patients attending the outpatient clinic were screened by their treating physician using the surprise question: ‘*Would I be surprised if this patient were to die within the next 12 months*?’ Patients were eligible if the treating physician answered *‘no’* to the surprise question. Subsequently, treating physicians were encouraged to inform and invite patients for an ACP conversation. If a patient agreed to participate, an information folder and a preparatory questionnaire was provided, and an ACP conversation was scheduled at the outpatient clinic. A trained nurse practitioner or specialised nurse and the treating physician facilitated the ACP conversation. Patients’ proxy/proxies were encouraged to participate in the conversation. Since the conversations were part of a feasibility study, in several of the conversations a member of the research team joined to observe the conversation and assist if needed. Two members of the research team were physicians working at the intensive care unit (ICU). The ACP conversations were audio recorded, transcribed verbatim and pseudonymised.

### Analysis of ACP conversations: a narrative approach

For the qualitative analysis of the ACP conversations, we chose to take an alternative approach to conducting a thematic analysis. A potential drawback of thematic or content analyses is that they sacrifice depth and context in the process of collating and generalising the content of individual interviews [9]. Roest *et al*. [9] provide a practical template for a narrative analysis aimed *‘at fostering a more in-depth understanding of the persons and practices being studied’* and *‘helping researchers trained in medicine and bioethics access valuable insights and techniques from interpretivist traditions’*. According to this proposed narrative approach, a systematic analysis [10] can be enriched by paying attention to narrative features and contextual levels in order to better understand *what* is said in qualitative interviews, *how* and in *what context* [9]. ACP conversations are complex conversations since various topics are discussed between patients, proxies and healthcare professionals. In order to honour and maintain the richness and variety of these the conversations, we adapted the narrative approach of Roest *et al*. [9] for this study.

### Creating a template for the narrative summaries

Our analysis began with us familiarising ourselves with the data, identifying primary areas of interest related to person-centred care and creating a template for the narrative summary (Appendix 1). The template for the narrative summary used for analysing the ACP conversations consisted of a series of questions aimed to guide analysis by capturing the essence of the conversation in a way that was both thorough and efficient. The research team consisted of three experienced qualitative researchers (EvL, JvD and MM) and two medical students (LI and MR). Four of the researchers (EvL, LI, MR and JvD) have a medical background. Moreover, JvD is professor in medical ethics and MM is associate professor in narrative medicine.

### Analysing the narrative summaries

A narrative summary was created and analysed for each ACP conversation in the MUTUAL feasibility study. During this phase the research team had regular meetings to discuss and compare the independently created narrative summaries of various ACP conversations. This process of iterative comparison allowed for discussions of different interpretations of the transcripts and discussion of potentially important emerging topics.

We added narrative vignettes to illustrate what happens within an ACP conversation [11]. This is a method ‘*to portray the actions of particular persons in specific events or of quotes of what particular persons said in various interviews […]*’ as described by Erickson [12, 13]. Names and personal details within the narrative vignettes are modified to ensure anonymity.

The study was assessed by the institution’s ethical review board at Gelderse Vallei Hospital, who judged this study was outside the scope of the Dutch law on research involving humans. Patients participating in the study provided written informed consent for participation and audio-recording of the conversation.

## Results

### Baseline characteristics

In total, 20 ACP conversations took place during the feasibility study (10 at the geriatrics department and 10 at the pulmonology department). Two conversations were not audio recorded due to technical issues and could not be included in the analysis. In 17/18 conversations a physician joined. Four nurses, one geriatrician and three pulmonologists participated in the ACP conversations. Patient characteristics are presented in Table 1. Total duration of the audio-recordings was 17 h and 4 min. Mean duration was 57 min (range 39–83). Mean duration of the first part of the conversation (with the nurse) was 39 min (range 24–48) and the mean duration of the second part (with the nurse and physician) was 16 min (range 6–25).

**Table 1:** Patient characteristics

	**Total (*n* = 18)**	**Geriatrics (*n* = 9)**	**Pulmonology (*n* = 9)**
*Mean age, in years (range)*	71.8 (49–91)	77.0 (65–91)	66.6 (49–76)
*Female sex (%)*	12 (67%)	7 (78%)	5 (56%)
*Primary diagnosis (n)*		Parkinson’s disease (*n* = 3) Dementia (*n* = 3) Mild Cognitive impairment (*n* = 2) Severe osteoporosis (*n* = 1)	COPD Gold IV (*n* = 5) Lung cancer (*n* = 4)

### Strategies for person-centred care within ACP conversations

We noticed variations in person-centred and treatment-centred focus within the ACP conversations. In all MUTUAL ACP conversations, shifts between discussing aspects of person-centred care and discussing future medical treatments occurred, both in the first as in the second part. Healthcare professionals used various techniques to explore what was important to the patient. This included asking about important values, goals and preferences, asking clarifying questions and checking whether something was understood correctly. Active listening strategies and allowing space for emotions and silence within the conversations were also noted within the conversations. In our analysis of the ACP conversations, we identified three important strategies that may enhance more person-centred ACP conversations. These strategies will be discussed more in depth below and are supported by narrative vignettes.

### Accepting that ACP is an individual process

We found that every patient was in a different phase within his/her ACP process. Some patients had already discussed their treatment preferences with proxies, some patients had not thought about their preferences regarding end-of-life care and others had thought about it but were not able to concretise their preferences. Aspects of person-centred care as well as preferences for future medical treatments in certain scenarios (such as worsening of the underlying condition, resuscitation, ICU admission etc.) were addressed by the facilitating nurse in the first part of the conversation, as well as in the second part of the conversation with the physician. Tailoring the conversation to where the patient is within the ACP process could help maintain the focus on discussing person-centred care. To do this, we believe it is important that healthcare professionals recognise and accept where the patient is in his or her individual ACP process. Most healthcare professionals explicitly stated that the conversation was part of a process and that patients were not expected to make any concrete choices. This reassurance may help to continue the conversation about what matters to the patient. Patients were also encouraged to continue this process after the conversation. Vignette 1 illustrates that recognition and acceptance of where the patient is in the process may create space for exploring what matters most to the patient, which is of utmost value in supporting the patient in the decision-making process. Sometimes, facilitators had difficulties accepting where the patient was in the process and focused too much on concrete treatment preferences. Focussing on medical aspects without first exploring what is important to the patient could prevent the patient from expressing him/herself.

**Vignette 1—seeing ACP as a process and acceptance of where the patient is within this process**

Susan is a 78-year-old patient; she is married and has four children. She lives at home, together with her husband. She describes her physical well-being as ‘good’; however, she is not able to handle unexpected situations. She states that she does not think about the future and that she has not written any advance directives. She adds: ‘*We’ll see when we get there’.* During the first part of the conversation, the nurse states twice that talking about treatment preferences is a process, and that the conversation they are having is part of this process. The nurse also explains that there is no need to document specific treatment preferences at the end of the conversation. During the conversation, it becomes clear what is important to Susan in her life. When the nurse asks whether she fears death/dying, the patient states ‘*No. I have faith*’. Subsequently, the nurse asks whether her religion gives support, which the patient then confirms. They discuss several medical scenarios, such as hospital admission after a collapse, resuscitation and ICU admission. Susan is not completely sure what her preferences are for future medical treatments. However, both Susan and her husband agree that extension of life is not preferred when nearing the end of life. She wants to avoid suffering at the end of life at all costs, and she states: *‘It is better to reconcile oneself to it [the end of life] ’.* Susan adds that it will really depend on her physical functioning and prognosis. The physician joins and the nurse gives a summary of the conversation. They all agree not to document specific treatment restrictions for now. At the end of the conversation, the nurse suggests they continue to talk about this at home.

The nurse and the physician do not try to convince the patient to decide on certain treatment restrictions. At the same time, they provide information concerning several topics, such as prognosis and the impact of admission to the ICU. This helps Susan broadly formulate her goals of care and could prove helpful in the decision-making process in a later stage.

### Exploring the underlying motivation for treatment preferences

As stated before, many shifts between discussing aspects of person-centred care and discussing future medical treatments occurred during the conversations. These shifts occurred in various ways: naturally, by either the patient, proxy or healthcare professional, or prompted by the healthcare professional. In all conversations, treatment preferences were already discussed by the nurse before the physician joined. Both nurses and physicians focussed on technical aspects of treatments, often explaining what resuscitation or admission to the ICU would entail. Physicians often emphasised the importance of discussing and documenting treatment preferences, irrespective of the nature of the decision. In most conversations, exploring the underlying motivation for certain treatment preferences gave insights into the patient’s wishes, values and needs and discussing treatment preferences served as an avenue to elaborate on patient values and preferences for future goals of care. For instance, the rationale for a patient’s preference not to be resuscitated can provide useful person-centred information for future decision-making. Vignette 2 gives insight into how the patient experiences his deteriorating health, what matters most to him and what his preferences are for future ICU admittance. The vignette shows that it is important to discover the patient’s treatment preferences and underlying motivation as a means of facilitating person-centred care discussions. It also shows that it is essential for healthcare professionals to check their understanding of the patient’s preferences. Within vignette 2, the nurse discovers the patient’s underlying preferences for ICU admission because of her curious attitude and wish to understand the patient’s motivation. Exploring the rationale, reformulating and summarising the patient’s preferences are effective means of checking whether the patient’s wishes are understood in order to achieve more person-centred ACP conversations.

The conversation summary given by the nurse to the physician often helped to verify and clarify patient wishes and to gain deeper insight into these wishes. Moreover, the conversation summary may help the physician joining the conversation gain a better understanding of the patient's values and preferences. Most summaries referred to various aspects discussed during the first conversation, including the patient’s most important values, worries and fears. Additionally, the patient’s treatment preferences were mentioned and, often included factors influencing the decision-making process, referred to as ‘procedural factors’.

**Vignette 2—example of the importance of exploring the rationale for treatment preferences**

William, an 84-year-old patient participates in the ACP conversation together with his niece. During the first part of the conversation, it becomes clear that maintaining his independence and not being a burden to others is of utmost importance to him. When exploring his preferences for future care, he explicitly states that he does not want to be admitted to the ICU: ‘*Care at an intensive care unit, which is a form of special care, can diminish your disease, but you can also die. Well, let me die then*’ . A few minutes later, he states the opposite by saying that he would want to stay at the ICU for a maximum of 4 weeks. The nurse notices this incongruence and tries to understand the patient by reformulating her question on his preference for ICU admittance. The patient then answers: ‘*That is not the point. I want to [conserve] what is still functioning in my body, not my eyes, definitely not my ears.*’ Now the nurse understands that he is talking about donating his organs and they discuss this further.

During the conversation it turns out that William wishes to donate his organs after he dies, and that he wants to be admitted to the ICU to adequately preserve his organs.

### Strive for an adaptive, curious and engaged attitude throughout the ACP conversation

We identified several patterns that may facilitate or hamper person-centred care discussions. A facilitating pattern consisted of certain techniques used by the facilitator that resulted in more detailed and concrete answers from the patient. These included asking a mix of open questions, clarifying questions and follow-up questions. This allowed the facilitator to gain a deeper understanding of the patient’s thoughts regarding several topics. For instance, asking open questions on topics such as their quality of life, course of their disease and goals or fears for the future appeared to encourage patients to express their values; asking clarifying questions allowed for more comprehensive reactions from patients and helped concretise the patient’s thoughts when symbolic language was used; asking follow-up questions allowed for deeper understanding of the patient’s perspective. Additionally, giving short summaries, verifying whether the facilitator understood the patient correctly, providing examples and involving proxies also seemed to facilitate person-centred care discussions.

Furthermore, our analysis indicates that healthcare professionals who demonstrated an adaptive, curious and engaged attitude achieved more person-centred ACP conversations. In most conversations, healthcare professionals were already familiar with the patient. In some conversations, healthcare professionals assumed they were aware of the patient’s treatment preferences due to their existing professional relationship. ACP conversations are an opportunity to get to know and understand the patient better. Vignette 3 describes how a physician enters an ACP conversation, has the opportunity to get to know the patient better and misses this opportunity assuming he already knows the patient and her treatment preferences.

Exploring the patient’s emotions, fears and worries facilitated person-centred care discussions within the conversations. Sometimes, the opportunity to do this was missed. For example, a patient gave several cues during the conversation about what was on their mind. Missing one cue was not identified as problematic. However, when this happened several times throughout a conversation it may have resulted in missed opportunities for obtaining information on the patient’s most important values. Sometimes emotions were acknowledged without elaborating why something was difficult or emotional. Instead of addressing emotions, fears and/or worries, facilitators sometimes switched topics, gave practical solutions or solely focused on preferences for medical treatments. This is illustrated in vignette 4 where the physician does not allow the relative to continue her story and does not address her fears. Instead, the physician immediately starts giving practical (medical) solutions.

**Vignette 3—example of not having a curious attitude**

Olivia, a 61-year-old patient, diagnosed with COPD, participates in the ACP conversation by herself. She discussed the preparatory questionnaire with her husband in preparation for the conversation. Within the first part of the conversation, she states that being independent is very important to her. She describes in detail what a good day and what a bad day looks like for her. She is aware of the deterioration of her health, and she accepts this. However, the condition of her lungs frightens her. She becomes very emotional when she talks about her father’s death, explaining that this is not how she wants to die. During the conversation, the nurse and Olivia also discuss her concern that her husband will not be able to take care of himself in case she dies. Olivia explicitly states that she only wants to be admitted to the hospital if she has a chance to recover and go home. Otherwise, she prefers to die at home. In the second part of the conversation, the physician joins. The nurse gives a summary of the conversation. The physician interrupts and says ‘*Of course, we have already discussed about […] what you want and what you don’t want any more […] Is there anything new? New to me? Or things that you think we have not discussed yet?’* This is directly followed by referring to the advance directives. Almost directly followed by *‘Excellent. I think this is very clear, do you have any questions?’*

The physician avoids being informed about topics Olivia discussed with the nurse during the first part of the conversation. This prevents the physician from getting to know the patient better and understanding the rationale for her treatment preferences, what is important to her and what she worries about.

**Vignette 4—example of a missed opportunity to discuss patient’s most important emotions, fears and/or worries**

Benjamin, a 67-year-old patient diagnosed with COPD, participates in the ACP conversation with his wife. In the second part of the conversation, the nurse gives a summary of the conversation. Within this summary, the nurse addresses that Benjamin is sometimes very short of breath and this results in a stressful situation for Benjamin and his wife. Subsequently, the nurse addresses the question that arose during the first part of the conversation: *‘What is the right moment to call in case Benjamin is short of breath?’* Benjamin’s wife starts explaining their question: *‘Last Thursday it suddenly happened. In the morning at 5 a.m. Benjamin sat up at once, incredibly short of breath. It is hard to get in contact with him, he is not able to talk. But he doesn’t want me to call, but…’* The physician interrupts and says *‘No, what we should do is create an emergency plan. That means that you have a list of things you can do [in case of emergencies]. That could also be a little bit of liquid morphine or a pill under the tongue with morphine, to take away the feeling of shortness of breath*.*’*

Benjamin’s wife continues stating how scary the situation was, but the physician interrupts her to give practical, medical advice.

A pattern that may hamper person-centred care comprised of too much focus on discussing treatment preferences, potentially resulting in reduced exploration of personal values. For example, in some conversations the facilitating nurse and/or physician focused on discussing treatment preferences at the cost of discussing patient’s values and future goals. For example, if patients were not able to formulate their preferences for future goals of care concretely, the facilitating nurse and/or physician continued asking questions about specific treatments.

Lastly, some techniques, such as drawing conclusions, could be either facilitating or obstructing to person-centred care discussions. If the patient had difficulties expressing himself or herself, drawing tentative conclusions or providing examples could support the conversation. This sometimes resulted in obtaining information on the patient’s values and preferences for care, hereby facilitating person-centred care discussions and fostering patient autonomy. For example, in one conversation the nurse assumed that filling out the questions from the questionnaire was confrontational. The patient agreed with her. The nurse then continued to talk about the patient’s disease course and previous hospital admissions, assuming that being admitted to the hospital several times must have been difficult and checking whether her assumption was right. In this case, the patient’s responses to the facilitator’s articulated assumptions revealed a lot about what she valued and how she was affected by her disease.

On the other hand, drawing a presumptuous conclusion can sometimes terminate the conversation on a specific topic, potentially depriving the patient of the opportunity to formulate his/her thoughts. For example, in one conversation the patient’s proxy stated that the patient was often emotional at home. The nurse replied that this was probably due to ‘her life’; she did not ask more about these emotions, and changed the topic. Asking what had made the patient so emotional could have given more insight into what the patient and her proxy considered important.

## Discussion

This study aimed to explore variations between person-centred care discussions and treatment-centred care discussions within ACP conversations in the MUTUAL intervention and how person-centred care discussions could be encouraged. We noticed variations in person-centred and treatment-centred focus within the ACP conversations. In our dataset we found shifts between person-centred care and treatment-centred care topics throughout the conversations. The structure of the MUTUAL intervention provides room for both person-centred care discussions and opportunities for discussing and documenting treatment preferences. The first part of the ACP conversation is where the relational work starts as referred to by Bulli *et al*. [14], and let healthcare professionals get to know the patient as a person. We identified three important strategies for ACP conversations to encourage person-centred care discussions within ACP conversations.

First, we believe it is important that healthcare professionals recognise and accept where the patient is within his or her individual ACP process. Not making decisions right away can also be part of a decisional process. Barnes *et al*. [15] conducted an analysis of dialogues between patients with advanced cancer and care planning mediators. They also highlight the importance of accepting and respecting in case a patient is not ready to discuss certain topics. Zwakman *et al*. [16] describe that readiness for ACP is not static, but rather something that can grow during an ACP conversation and can change over time. We found something similar with respect to treatment preferences: often patients stated within the ACP conversation that they had not thought about treatment preferences or that they did not have specific treatment preferences, whereas later in the conversation it turned out that they had thought about specific situations already or were able to formulate their preferences during the conversation. Thus, patients can ‘become’ ready to discuss treatment preferences, just as patient’s treatment preferences can gain in clarity within an ACP conversation.

Secondly, it is important to explore the underlying motivation for patient’s treatment preferences. Discussing treatment-centred topics can serve as an avenue to elaborate on patient values and preferences and hereby foster patient-centredness within ACP conversations. Quill *et al*. [17] found that when patients are asked about setting limits for medical treatments and they respond with ‘doing everything’, this answer may be less about medical decisions and more about fears concerning their future health/sickness. They state that focusing on exploring patient wishes, followed by focusing on what appropriate care entails could prevent misunderstanding and conflict when discussing preferences for end-of-life care. Hence they encourage healthcare professionals to explore the underlying reasons [17]. This is in line with our results.

Lastly, healthcare professionals who demonstrated an adaptive, curious and engaged attitude throughout the ACP process achieved more person-centred ACP conversations. In our study, missing opportunities to discuss a patient’s emotions, fears and/or worries was identified as a limiting factor to achieve person-centred care discussions. Ahluwalia *et al*. [18] reported that 84% of the opportunities were missed when patients expressed concerns, questions and thoughts regarding their future care by physicians during regular outpatient clinic visits. Briedé *et al*. [19] reported that only 3.1% of the consultations at the internal outpatient clinic involved discussions of future care, increasing to 17.6% after receiving training on the importance of discussing preferences for care. Briedé *et al*. [19] suggest that listening actively and carefully to the patient and encouraging them to speak and form their own conclusions should be encouraged.

### Implications for practice

This study has concrete implications for practice since it gives concrete strategies that may encourage more person-centred ACP conversations. Accepting where the patient is within the ACP process may enable a conversation more focused on what is important to the patient and may help in the future decision-making process, even if the patient has not explicated any treatment preferences. We showed that exploring the patient’s rationale for treatment preferences and not only the treatment preferences themselves can give insight into patient’s wishes, values and needs. These rationales should also be shared with other involved healthcare professionals. Decisions in the acute care setting are not straightforward. For instance, reasons for admission to an ICU are various and a patient’s prognosis heavily depends on the reason for admission (multiple organ failure is different compared with urosepsis). It is not possible (or preferable) to discuss all potential medical scenarios. We contend that discussing what is important to the patient and knowing the rationale for treatment preferences can be helpful in decision-making, even in future situations that have not been specifically addressed during an ACP conversation.

### Strengths and limitations

This study analysed the content of ACP conversations of a recently developed ACP intervention: the MUTUAL intervention. Strength of this study is that 18 audio-recordings and transcripts of actual ACP conversations were available for analysis. This enabled studying the interaction between patients, proxies and healthcare professionals. This may help healthcare professionals in conducting ACP conversations that focus both on person-centred care and medical decision-making. Moreover, our narrative approach allowed for a deeper understanding of what is discussed in these conversations, how and in what context. This approach, combined with our research team with different backgrounds, led to in-depth discussions on the interpretation of the ACP conversations. Although this study gives several insights, it also has some limitations. First, the feasibility study only included patients and healthcare professionals from the pulmonology and geriatrics department, limiting the generalizability of our study. Secondly, the included ACP conversations are part of a feasibility study. We have not been able to investigate potential differences in the experiences of nurses and physicians when conducting ACP conversations. Finally, during several conversations, a member of the research team was present, potentially influencing the conversation.

## Conclusion

We noticed variations in person-centred and treatment-centred focus within the ACP conversations in the MUTUAL intervention. Certain strategies by healthcare professionals facilitated a more person-centred focus. This includes tailoring the conversation to where the patient is within the ACP process, exploring the underlying motivation for preferences and remaining curious throughout the ACP process.

## Supplementary Material

aa-23-0869-File002_afae020
