# Supplementary material for: Finding the balance between person-centred and treatment-centred discussions in advance care planning—a qualitative analysis of conversations within the MUTUAL (Multidisciplinary Timely Undertaken Advance Care Planning conversations) intervention using a narrative analysis
Source: Age Ageing. 2024 Mar 6;53(3):afae020. doi: 10.1093/ageing/afae020 (PMC10919783; doi:10.1093/ageing/afae020)
Supplement: aa-23-0869-File002_afae020 [file aa-23-0869-file002_afae020.docx]

# Appendix 1 – Narrative summary template

| **General information** | |
| --- | --- |
| Name researcher |  |
| Date ACP-conversation |  |
| Information those present (patient, proxies, research team, etc..) |  |
| Information patient (age, gender, underlying diagnosis, reason for ACP-conversation…) |  |
| Information nurse (position, experience facilitating previous ACP-conversations…) |  |
| Information physician (position, experience facilitating previous ACP-conversations …) |  |
| **Summary** | |
| Short summary of the **first part** of the ACP-conversation (*balance person-centred discussions and treatment-centred discussions)* |  |
| Short summary of the **second part** of the ACP-conversation (*balance person-centred discussions and treatment-centred discussions, what are the most important considerations/ arguments for certain treatment preferences/restrictions)* |  |
| To what extent is the conversation summary by the nurse a good representation of the ACP-conversation? And why?   - Are all the discussed elements mentioned? - Is the process described/explained? (“procedural factors”) - Is the conversation summary beneficial for the second part of the conversation? Is it important? |  |
| To what extent are the agreed treatment preferences/ restrictions a good representation of the ACP-conversation? (*E.g. based on the human, illness or information?)* And does the physician accept these preferences/agreements? |  |
| What are the most important values to the patient? Give a representative quote which shows this |  |
| What is the balance between person-centred discussions and treatment-centred discussions in the ACP-conversation? Describe the one or two most important shifts between person-centred discussions and treatment-centred discussions:   - *When?* ***(When)*** - *By whom?* ***(Who)*** - *Why/what for?* ***(What for)*** - *How: logical continuation or guided?* ***(How does it feel)*** - *What does it yield?* |  |
| Describe the two most facilitating and obstructive factors for reaching an in-depth understanding of person-centred discussions and treatment-centred discussions (provide two short clarifying quotes if needed)   - *Examples of facilitating factors for person-centred discussions: asking about worries, goals, preferences, important values, asking follow-up questions, reformulating questions, showing interest (e.g. signs of active listening), checking if he/she understood the patient, identifying and addressing emotions, involving proxies, signs of creativity/flexibility (e.g. deviate from the interview guide), tailoring information to the patient* - *Examples of obstructive patterns for person-centred discussions: too much focus solely on treatment-centred discussions, negative influence from proxies/ healthcare professionals, not using facilitating factors* |  |
| To what extent does the preparatory questionnaire play a role in the ACP-conversation? *(E.g. how is the preparatory questionnaire filled out, how did the patient/proxies experience the preparation, does the preparation result in an in-depth conversation, considering the difference in effect of the preparation before and during the conversation?)* |  |
| What role does the nurse play in the ACP-conversation? *(E.g. listener, expert, translator,…)* |  |
| What role does the physician play in the ACP-conversation? *(E.g. listener, expert, translator,…)* |  |
| Describe the ambiance during the conversation  *(Difference between the first and second part, what role does emotion play in the conversation?)* |  |
| Are there sudden changes? *(E.g. change of perspective, using the I/we form)*  Are there different storylines?  Are there any ambiguities? |  |
| **Narrative features** | |
| What are the explicit and implicit fears, worries and barriers?   - *Of the patient?* - *Of proxies?* |  |
| Describe the use of metaphors/symbolic use of language *(How is referred to death/nearing the end-of-life? Is this concretized?)* |  |
| What grade would you give the collaboration in the conversation on a scale of 1-10?   - *Between the patient/proxies and healthcare professional?* - *Between the healthcare professionals among themselves?* |  |
| **Annotations researcher** | |
| Impressions and observations during listening/reading? |  |
| Emerging questions during listening/reading, which could potentially contribute to an extensive interpretation/analysis |  |
| Other remaining observations |  |
